# Effectiveness of a behavioral treatment protocol for selective mutism in children: Design of a randomized controlled trial

**DOI:** 10.1016/j.conctc.2020.100644

**Published:** 2020-08-12

**Authors:** C. Rodrigues Pereira, Judith B.M. Ensink, Max G. Güldner, Kees J. Kan, Maretha V. de Jonge, Ramón J.L. Lindauer, Elisabeth M.W.J. Utens

**Affiliations:** a Amsterdam UMC, University of Amsterdam, Department of Child and Adolescent Psychiatry, Amsterdam Public Health, Amsterdam, the Netherlands; b Academic Center for Child and Adolescent Psychiatry the Bascule, Amsterdam, the Netherlands; c Research Institute of Child Development and Education, University of Amsterdam, the Netherlands; d Institute of Pedagogical Sciences, Leiden University, the Netherlands; e Department of Psychiatry, UMC Utrecht, the Netherlands; f Department of Child and Adolescent Psychiatry/Psychology, Erasmus MC – Sophia Children's Hospital, Rotterdam, the Netherlands

**Keywords:** Selective mutism, Anxiety, Children, Behavioral therapy, Treatment at school, DSM, Diagnostic and Statistical Manual of Mental Disorders, RCT, Randomized Controlled Trial, SMQ, Selective Mutism Questionnaire, SM, Selective Mutism, WCG, Waiting List Control Group

## Abstract

Selective mutism (SM) is a relatively rare anxiety disorder, characterized by a child's consistent failure to speak in various specific social situations (e.g., at school), while being able to speak in other situations (e.g., at home). Prevalence rates vary from 0.2% to 1.9%. SM is usually identified between the ages of 3–5 years. It is often underdiagnosed and consequently children receive no or inadequate treatment, with negative consequences for school and social functioning. If left untreated, SM can result in complex, chronic anxiety and/or mood disorders in adolescence and impaired working careers in adulthood. Currently, no evidence-based treatment for SM is available in the Netherlands, therefore this study aims to [1] test the effectiveness of a treatment protocol for SM that is carried out at school, and to [2] identify baseline predictors for treatment success.

This article presents the design of a randomized controlled trial into the effectiveness of a behavioral therapeutic protocol for selective mutism in children (age 3–18). The expected study population is n = 76. Results of the treatment group (n = 38) will be compared with those of a waiting list control group (WCG) (n = 38). Pre and post treatment assessments will be conducted at comparable moments in both groups, with baseline assessment at intake, the second assessment at 12 weeks and post-assessment at the end of treatment. If proven effective, we aim to structurally implement this protocol as evidence-based treatment for SM.

## 1 Background

Selective mutism (SM) is a severely debilitating anxiety disorder, mostly occurring in young children [1]. It is characterized by consistent failure to speak in situations where speaking is expected (e.g., in school), while speaking freely in other situations (e.g., at home). Prevalence rates vary between 0.2% and 1.9% [2,3], with a boys:girls ratio of 1:2 [3]. SM is more common in bilingual or multilingual children [4]. Not speaking in social situations impairs personal, social, and academic development. If left untreated, SM can have a chronic character [5,6].

Currently, it is still unclear which mechanisms affect treatment success in children with SM. For childhood anxiety disorders in general, it is known that parental factors (parenting style, parental psychopathology and previous health care consumption) can influence treatment success [7]. A study of Oerbeck, Stein [8] shows that children who are treated for SM at a younger age, show faster treatment progress than older children. These results were maintained on longer term [9]. In another study of Oerbeck, Overgaard [10], besides age, higher severity at baseline, familial anxiety and compliance of parents were predictors of treatment success. Also the predictive value of bilingualism, gender, comorbidity and minority status was investigated. These results were less compelling. As the authors also note, possibly due to a relative small sample size (N = 32) effect sizes were small. Especially at follow-up due to consequences of drop-out during the study. In our clinical institution we see a large, heterogeneous population of children with SM, with many bilingual children, as also described by Elizur and Perednik [4] and Toppelberg, Tabors [11]. Although several evidence-based treatments, based on (cognitive) behavioral therapy are available for anxiety disorders in children [12], studies investigating treatment effects in children with SM are still limited. A recent non-randomized trial showed that behavioral techniques with additional parent child interaction therapy improve SM symptoms [13]. Three previous randomized controlled trials (RCT) investigated showed that behavioral therapy was effective for selective mutism [8,14,15]. These studies provided therapy in different settings i.e. in the clinical setting [14,15] and at home before starting at school [8]. Unfortunately sample sizes in these studies are relatively small (N = 21, N = 24, N = 29). Currently, no evidence-based protocol specifically for the treatment of SM is available for the Dutch population. Since such a protocol is urgently needed in clinical practice, in our institution the protocol ‘Speaking in school, a matter of doing’ was developed [16]. A considerable strength compared to most previous studies is that this treatment is provided at school, where the problem is most present. The rationale is that treatment in school makes it easier to generalize speaking behavior to settings where the child still has difficulty speaking. In this RCT we aim to investigate the effectiveness of this behavioral therapeutic protocol, compared to a waiting list control group. A major methodological advantage is that we aim to execute a sufficiently powered RCT. Secondly, we aim to identify putative predictors for treatment success in children with SM.

## 2 Methods/design

***Design:*** this study is a single-center, single-blinded randomized controlled trial (RCT), comparing protocolized behavioral therapy for selective mutism to a waiting list control group.

***Inclusion:*** eligible are children between the age of 3–18 years old, with an estimated IQ of 85 or higher, who understand Dutch and are referred to our academic center for diagnosis and treatment of selective mutism between September 2018 and March 2020. This large age span covers all consecutive patients referred to our institution. Though the vast majority of these children is under the age of 8, we also get referrals up to 18 years.

***Exclusion******:*** ineligible are children who have primary diagnoses other than selective mutism. Comorbidity is not excluded, but the primary and most impairing diagnosis has to be selective mutism. During the intake, it will be assessed what the most urgent need for help is and what type of care is most indicated. In the case that treatment is indicated for a comorbid diagnosis at first, the child will be excluded from the study.

### 2.1 Patient recruitment and procedure

Parents of eligible patients will be personally informed about the research project by the psychologist-researcher and will receive written information about the research project. Written informed consent will be obtained from parents/guardians for patients under the age of 12. From the age of 12 written consent will also be obtained from them. Only patients (≥12 years), who have given informed consent, or whose parents have signed informed consent will participate in this study.

After informed consent is provided, children are randomly assigned to treatment for selective mutism (N = 38) or the waiting list control group (WCG: N = 38).

Randomization will be stratified for gender, age (children aged until 7 years old are stratified into the “young” group, and children ages 8 and up are in the “old” group) and bilingualism (speaking one language, i.e. Dutch, versus speaking multiple languages). Randomization will be performed by an independent researcher, who will inform the therapists regarding the randomization outcome.

### 2.2 Intervention

A standardized, behavioral therapeutic protocol was developed in the Netherlands to treat SM [16]. Children learn in different steps, by using gradual exposure to challenging situations, shaping and modelling. The advantage and innovative aspect of this protocol is that treatment is carried out solely at school. Since at school the problem is present most evidently, a therapist goes to school to practice individually with the child. This helps to generalize the learned exercises to the school situation. Every week the child learns step by step to make sounds and to speak, after which the speaking behavior is extended to various places in school with as the final goal: participating actively in the circle discussion in the classroom.

The protocol is mainly based on behavioral techniques, with as the primary aim stepwise practicing to speak. However, for children from 12 years and older, additional cognitive elements (e.g. helping thoughts) which are used in traditional cognitive behavioral therapy for anxiety [17], are available to be used optionally. The therapist involves the teacher as a co-therapist in the treatment process. After each weekly treatment session at school, the therapist instructs the teacher how profit can be maintained and which exercises to practice during the week. The teacher then continues the exercises throughout the week, finding short moments in time during the week to practice with the child. The therapist is in touch with the teacher face to face after each therapy session, by email or phone when necessary.

Previous clinical experience learns that the average treatment duration with this protocol is 22 weeks.

### 2.3 Assessments

Parents, children older than 8 years and teachers will complete questionnaires online. Parents will also be interviewed by the researcher.

The psychologist-researcher who performs the assessments will be blinded for participants’ allocated intervention. Children and parents, as well as the treatment-staff are explicitly asked not to talk about their randomization condition with the psychologist-researcher. Both groups are treated with the standard treatment protocol for selective mutism.

Assessments will be carried out at the following time points [1]: T1, baseline assessment during intake, before intervention starts [2]; T2, 12 weeks after baseline, either after first 12 weeks of treatment or 12 weeks of waiting list period [3]; T3, after last treatment session. If at any of the assessments or during the waiting list and/or treatment period there is sudden need for immediate intervention (e.g. in the event of a crisis), unblinding will take place to ensure that the participating patient receives the necessary care.

To acquire information from all informants (parents, guardians, teachers, children/adolescents), standardized questionnaires and interviews will be used with adequate psychometric properties, except for the Dutch translation of the SMQ, which will be validated in this study.

In Table 1, all assessments are listed.

**Table 1:** Measures per assessment moment.

Instrument	Variable	Assessment moment		
		T1	T2	T3
Primary outcomes				
Selective Mutism Questionnaire (SMQ)	Selective mutism symptoms	P	P	P
Selective Mutism section of Anxiety Disorders Interview Schedule (ADIS-C)	Selective mutism symptoms	P	P	P
Secondary outcomes				
ADIS-C	Anxiety and mood symptoms	P	a	P
Child Behavior Checklist (CBCL)	Behavioral and emotional problems	P	P	P
Teacher Report Form (TRF)	Behavioral and emotional problems	T	T	T
Youth Self Report (YSR)	Behavioral and emotional problems	C	C	C
Self-Perception Profile Child/Adolescent (SPP–C/A)	Self-image	C		C
Child Health Questionnaire (CHQ)	Quality of life	P		P
Predictors				
Adult Self Report (ASR)	Behavioral and emotional problems	P		
EMBU-P	Parenting style	P		
Demographic variables	Demographics	P		
Health care consumption questionnaire	Health care consumption	P		

P: Parents.

T: Teacher.

C: Child.

SMQ: Selective mutism questionnaire.

ADIS-C: Anxiety Disorder Interview Schedule – Children's version.

CBCL: Child Behavior Checklist.

TRF: Teacher Report Form.

YSR: Youth Self Report.

SPP-C/A: Self-Perception Profile Child/Adolescent.

CHQ: Child Health Questionnaire.

ASR: Adult Self Report.

EMBU-P: Egna Minnen Beträffande Uppfostran – Parent version.

a At T2, only the reported sections of the ADIS-C interview at T1 are assessed.

### 2.4 Primary outcomes

*Selective Mutism symptoms:* to obtain parent reports of selective mutism symptoms in their child, a) The Dutch translation of the Selective Mutism Questionnaire (SMQ) [18] and b) the selective mutism section of the Anxiety Disorders Interview Schedule [19] will be used.

For the purpose of this study, the Dutch SMQ will be validated. The SMQ consists of two scales, the symptom scale (17 items, response categories: “always”, “often”, “sometimes” and “never”), and the impediment scale (6 items, response categories: “no”, “moderate”, “fairly” and “a lot”). Within the symptom cluster, subscales for school, home and social environments are distinguished [18,20]. A higher score in the SMQ indicates more problems with speaking.

Validation of the SMQ will be based on the baseline scores (T1) of all children referred to our center for diagnosis and treatment of selective mutism.

### 2.5 Secondary outcomes

*Anxiety/mood symptoms*

Interview: The Anxiety Disorders Interview Schedule (ADIS-C) clinical interview is used to obtain information from parents about possible comorbid problems.

Questionnaires: Emotional and behavioral problems will be assessed by the following questionnaires, which have parallel items.

The Child Behavior Checklist (CBCL) [21,22] will be used to obtain standardized parent reports of emotional and behavioral problems in their child. The Youth Self Report (YSR) [21] is the parallel version of the CBCL, but items are formulated in the child form. Children of 11 years and older will complete the YSR.

The Dutch version of the Teacher's Report Form (TRF) [21,22] will be completed by the teacher of the child. The TRF assesses problem behavior (at school). In the CBCL, YSR and TRF questionnaires, a higher score indicates more emotional and/or behavioral problems. In these questionnaires, we are examining the internalizing and externalizing total scales as well as the subscales. The items related to (social) communication will also be examined individually.

*Self-worth image:* The Dutch version of the Self Perception Profile Child/Adolescent [23,24] is completed by children of 8 years and older.

*Quality of life:* The Dutch version of the Child Health Questionnaire (CHQ) [25,26] is used to assess quality of life of the child.

*Predictors*

*Parental psychopathology:* The Adult Self Report (ASR) [27] will be used to screen for parental emotional and behavioral problems.

*Parental parenting styles:* The Egna Minnen Beträffande Uppfostran – Parent version questionnaire (EMBU-P) [28] will be used to assess the parenting style parents use for the child.

*Demographic variables:* Demographic variables such as age, gender, socio-economic status and bilingualism will be assessed during intake, using a semi-structured interview used in care as usual.

*Health care consumption:* Previous physical and mental health consumption will be assessed during intake, using a standardized questionnaire [29].

*Treatment integrity*

To check treatment integrity, all treatment sessions will be audio/video-recorded (if consent of parents and child). Recordings will be saved safely and coded. A random 20% of all sessions will be rated by two independent raters. To avoid protocol drifting, monthly supervision will be given by a certified psychologist. The psychologists providing treatment are trained in the protocol.

*Sample size calculation*

A preliminary randomized clinical trial into behavioral treatment of selective mutism showed an effect size of Cohen's d = 0.58 on speaking in social situations [15]. To determine effects on our primary outcome (i.e. the pre versus post treatment change (T1-T3) on selective mutism symptoms), based on an effect size of d = 0.58 (medium effect size), an alpha of 0.05 (two-tailed) and a power of 0.8, a sample size of 76 (38 per group) is needed [30].

## 3 Statistical analysis

### 3.1 Possible difference between participants and drop-outs

Clinical experience in this patient population shows us that drop-out during treatment is rare. To be able to compensate for possible drop-out during the study (if needed), we aim to include 100 participants as a safety measure. A drop-out analysis on the drop-out population will be conducted: it will be studied whether there are possible differences in variables (such as: gender, age, socio-economic status, bilingualism, severity of selective mutism at baseline assessment and parental psychopathology) between the population that participates until the end of their treatment and the population that drops out. In this way it can be checked whether there is a selective dropout. A selective dropout can lead to selection bias, which could be statistically adjusted for.

### 3.2 Intention to treat

The statistical analyses will be based on the intention-to-treat principle. If appropriate, secondary analyses will be conducted using a per-protocol basis.

Primary and secondary outcomes: Pre-post changes (pretreatment and post treatment assessment of the treatment versus WCG group) on the primary outcome (symptoms of selective mutism) will be tested with repeated measures analyses (factorial repeated measures ANOVA). To determine pre-post change in treatment outcome (responders vs. non-responders) paired sample t-tests will be executed. The effectiveness on secondary outcome measures: symptoms of anxiety/depression, self-image, quality of life and care consumption, will be tested with the same analyses. With linear and logistic regression predictors (e.g., gender, age, socio-economic status, bilingualism, parental psychopathology and parenting styles) for treatment outcome can be determined (p < .05). In the analyses, the number of treatment sessions will be included as a covariate.

### 3.3 Validation of the Selective Mutism Questionnaire

The SMQ [18] is used in this study to assess symptoms of selective mutism. This questionnaire has not yet been validated for the Dutch population. In order to validate the SMQ, data will be used from baseline assessment of selective mutism symptoms in the children referred for diagnosis and treatment of selective mutism. In addition, data will be gathered from 240 healthy school children, recruited from elementary and secondary schools and sports clubs, from the same regional areas as the children with selective mutism. Control children will be adjusted to patients with a 3:1 ration. To assess the factor structure, confirmatory factor analysis will be applied. Split-half reliability and internal consistently (Chronbach's alpha) will be determined. Item-rest and inter-item correlations will be determined. The reliability of the items' response scale is calculated using Rasch assessment scale analysis [31]. To assess concurrent validity, results on the SMQ will be tested against outcomes on the selective mutism part of the Anxiety Disorder Interview for Children (ADIS-C).

## 4 Discussion

With the new classification of the DSM-5 [1], selective mutism is now categorized as an anxiety disorder. Selective mutism is often underdiagnosed which causes children with selective mutism to receive no treatment at all, inadequate or untimely treatment. In the Netherlands and Dutch speaking regions of Belgium, until now no evidence-based treatment for selective mutism was available.

Investigating the effectiveness of the treatment protocol for selective mutism is necessary to ensure that evidence-based treatment is available as standard care within mental health institutions for children and adolescents. The present treatment protocol has the advantage, that treatment takes place at school, where the selective mutism is often most present. The rationale is that once the child is able to speak at school, this makes it easier to generalize speaking behavior to other social settings. The protocol uses a behavioral therapeutic approach, which belongs to standard paradigm for the treatment of anxiety disorders, especially in children who are too young to profit from cognitive elements in treatment.

This study has several strengths. A standardized treatment protocol for behavioral therapy is used, with therapists trained in this intervention, with weekly supervision, and treatment integrity being checked. Treatment takes place in the school setting, enhancing generalizability of techniques learned. Importantly, we aim to identify predictors for treatment success, to get insight into which children benefit more or less form this treatment. This study aims to contribute to knowledge, by gaining insight into why some children improve more than others by investigating if demographic variables (such as bilingualism), parental psychopathology, parenting styles and previous health care consumption moderate treatment outcomes.

### 4.1 Limitations

Children are treated by trained therapists of a single center. Because there are relatively few children with selective mutism, the intended population might be heterogeneous in terms of demographics. This creates both an interesting opportunity for generalizability as well as a challenge in to what extent conclusions can be made from a heterogeneous study population. We aim to control for this by carefully assessing demographic variables to gain information about the characteristics of the population.

Treating children at school may provide logistic challenges (such as therapist's travel distance) but also creates positive opportunities in having a direct way of communication with the teacher about the exercises in the treatment and to be able to strengthen the motivation for participating in the treatment for busy teachers with full classrooms.

### 4.2 Implications for clinical practice

Since all referred children between three and eighteen years old will be screened, selective mutism can be detected early in development. Early treatment for selective mutism creates the potential to prevent future problems (secondary prevention), such as educational problems and the development of anxiety in adulthood. This project aims to identify an effective treatment protocol and unravel some of the underlying mechanisms of treatment success in children with selective mutism. Subsequently this will lead to improvement of care. We expect that the results of the presented study will be immediately relevant to clinical practice and that there is potential for large-scale roll-out across the Netherlands.

## Funding

This research project is funded by Fonds Stichting Gezondheidszorg Spaarneland (Grant ID: 2017284). The funding source had no role in the design of the study, and will not have any role in its execution, analysis, interpretation of the data, or decision to submit results.

## Availability of data and materials

Not applicable. This paper presents the study protocol and does not contain any data or results.

## Author’s contributions

All authors critically reviewed the manuscript for intellectual content. All authors have read and approved the manuscript. CRP drafted the initial manuscript and submitted the manuscript for publication. CRP, JE and MG were responsible for study concept and design, which was supervised by RL and EU. CRP, JE, RL and EU were responsible for funding. MdJ provided intellectual input and feedback on the study design. MG developed the original treatment protocol and provided intellectual input for funding. KK was involved as statistician and provided information for the sample size calculation and the statistical analyses.

## Trial registration

Dutch Trial Registry: NTR7534

## Ethics approval and consent to participate

The Medical Ethics Committee of the Academic Medical Center approved this trial. This study will be conducted according to the Helsinki Declaration and its later amendments or comparable ethical standards. Informed written consents will be obtained from the parents or guardians of the participating children. This article does not contain any studies with animals performed by any of the authors.

## Consent for publication

Not applicable. This paper presents the study protocol and does not contain any data or results.

## Declaration of competing interest

The authors declare that they have no conflict of interest.
